# Supplementary material for: Fast and accurate single-cell RNA-seq analysis by clustering of transcript-compatibility counts
Source: Genome Biol. 2016 May 26;17:112. doi: 10.1186/s13059-016-0970-8 (PMC4881296; doi:10.1186/s13059-016-0970-8)
Supplement: Additional file 1 — Supplementary Figures. A PDF file that contains eight supplementary figures. (PDF 22937 kb) [file 13059_2016_970_MOESM1_ESM.pdf]

Supplementary Figures for

"Fast and accurate single-cell RNA-seq analysis by clustering of transcript-compatibility counts"

List of Figures

1: **Runtimes of bowtie, kallisto-quant, kallisto-pseudoalign, and computation of pairwise distance matrices.** 2

2: **More details on the 7 clusters obtained from affinity clustering in Trapnell *et al.*'s data-set.** 3

3: **More genes to validate the 3 clusters obtained from Trapnell *et al.*'s data-set.** 3

4: **Selecting parameters for affinity propagation on Trapnell *et al.*'s gene expression vectors** 4

5: **Quantifying after clustering to validate clusters obtained.** . . 5

6: **Comparison of different distance metrics to use to compute pairwise distances.** 6

7: **Comparing alignment-based TCC with pseudoalignment-based TCC** 7

8: **Selecting parameters for affinity propagation on TCC vectors for Trapnell *et al.*'s data-set.** 8

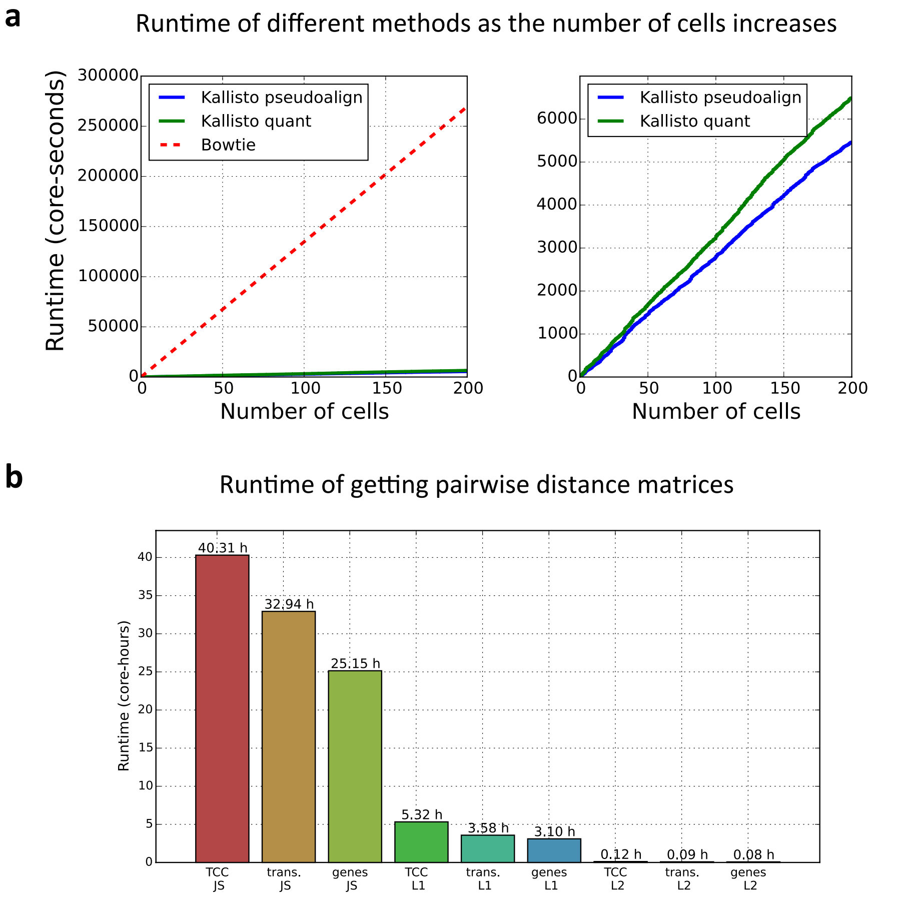

	TCC	transcripts	genes
Feature dimension	246981	82741	28250
Average # of non-zero entries	11804	8655	6747

Supplementary Figure 1: Runtimes of bowtie, kallisto-quant, kallisto-pseudoalign, and computation of pairwise distance matrices. (a) The runtimes of Bowtie, kallisto with both pseudoalignment and quantification (kallisto-quant), and kallisto with just pseudoalignment (kallisto-pseudoalign) were obtained for 200 randomly selected cells from Zeisel *et al.*'s 3005 mouse brain cell dataset [1] as shown on the left pane. The (extrapolated) runtime of Bowtie was higher than the runtimes of the two pseudoalignment-based methods. When comparing kallisto-quant against kallisto-pseudoalign (as shown on the right pane), kallisto-pseudoalign is slightly faster, saving approximately 5 seconds per cell. As the number of cells scales up to $44,000$ for novel sequencing technologies such as DropSeq, kallisto-pseudoalign will have savings of about 60 hours compared to kallisto-quant and 1.8 years compared to bowtie. **(b)** The runtimes obtained for running pairwise distances on the distributions obtained from TCCs, transcriptome expressions, and gene counting are shown here. These times are shown for Jensen-Shannon distance and ℓ_1 distances. The feature dimension indicated in the table equals the number of features (either TCC, transcript abundances, or gene abundances) that are non-zero in at least one of the 3005 samples.

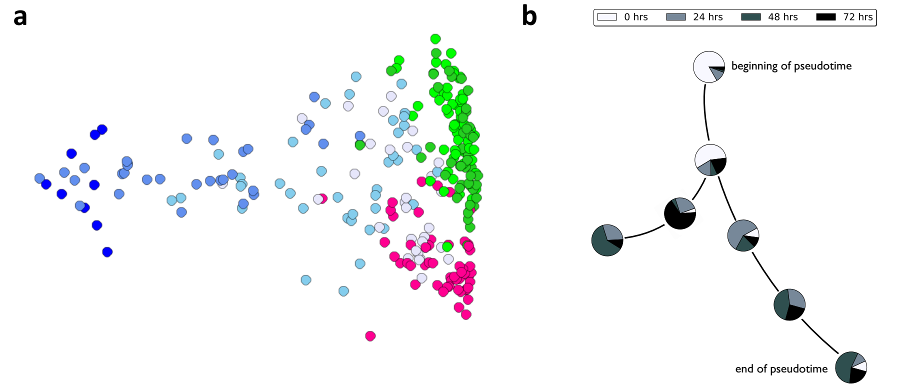

Supplementary Figure 2: More details on the 7 clusters obtained from affinity clustering in the data-set of [2]. (a) Shows the diffusion map of cells colored by the labels of the 7 clusters. **(b)** Each pie-chart node in the MST shows the distribution of the cells of each cluster in real-time. The tree on which these are placed corresponds to the pseudotime obtained.

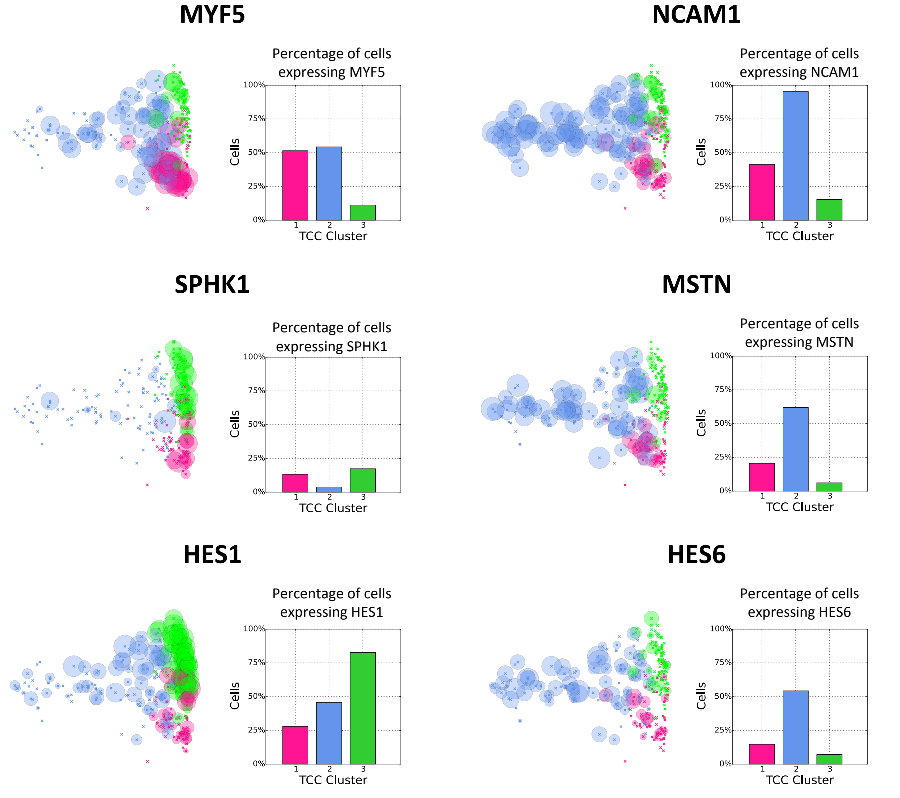

Supplementary Figure 3: More genes to validate the 3 clusters obtained from Trapnell et al.'s data-set [2] Shows the distribution of various other genes that are known to be markers of the three states represented by the three TCC clusters. The patterns discovered here using TCC closely matched those found by Trapnell *et al*.

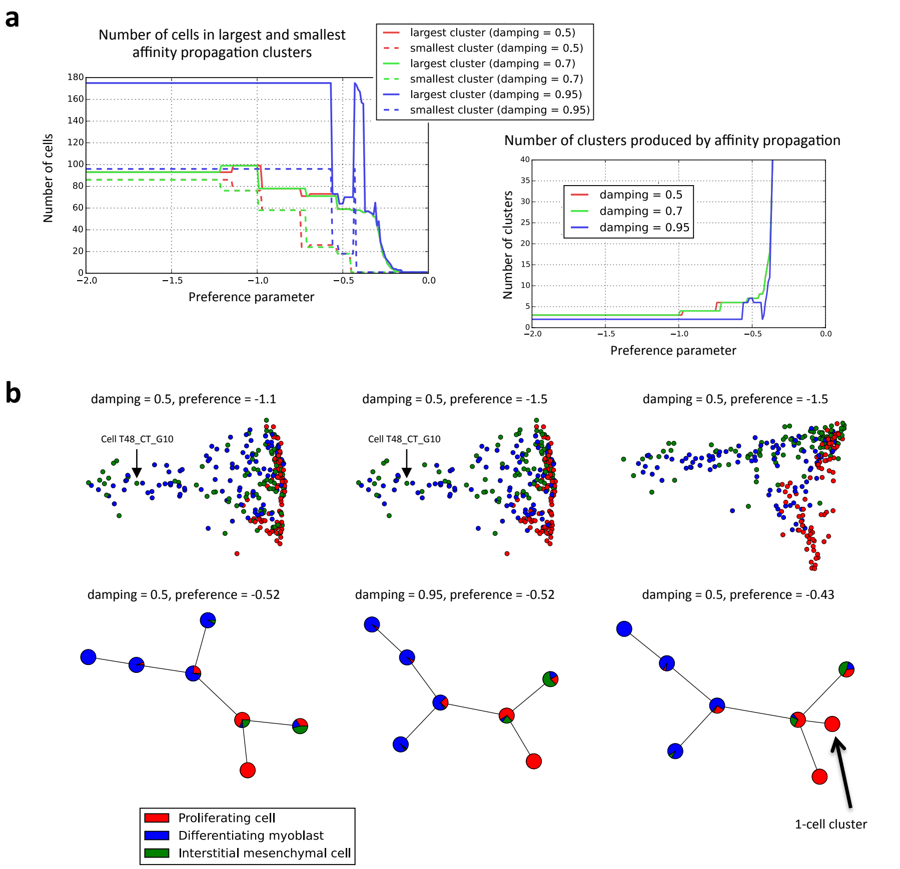

Supplementary Figure 4: Selecting parameters for affinity propagation on [2]'s gene expression vectors We note that choosing optimal parameters for affinity propagation requires some biological intuition. **(a)** For each of 3 damping parameter values, we swept through multiple preference parameter values. We looked for a combination of parameters that produced a reasonable amount of clusters roughly the same size. The left plot show two curves for each damping parameter: a dotted one indicating the number of cells in the smallest cluster and a solid one indicating the number of cells in the largest cluster. In the case where we do not know the correct number of clusters, we would use clusterings immediately before the large spike in number of clusters (right plot), resulting in about 7 clusters. The plots shown here are generated using Trapnell et al.'s expression vectors. We also noticed that from empirical testing, varying parameters in a flat region of the plot resulted in the exact same clusters. **(b)** There are multiple combinations of parameters that could generate 3 or 7 clusters. Here we use Trapnell et al.'s expression vectors to generate two MSTs. Each MST uses one of two combinations of damping and preference parameters selected based on the plots in (a). Slight tweaking of the preference parameters can result in an MST with 8 clusters, as shown in the right-most tree. Like we did in Figure 4, we would collapse the 1-cell cluster into its nearest cluster. Knowing that 3 cell types exist in the population, we also tried another two combinations of parameters to produce 3 clusters. For easy comparison to the TCC results in the main text, we visualized the clusters with the diffusion maps of Figure 4d. We see that the cell discussed in Figure 5 (T48_CT_G10) still fails to be classified as a differentiating myoblast. For additional comparison, we computed another diffusion map using Trapnell et al.'s expression vectors (right-most diffusion map).

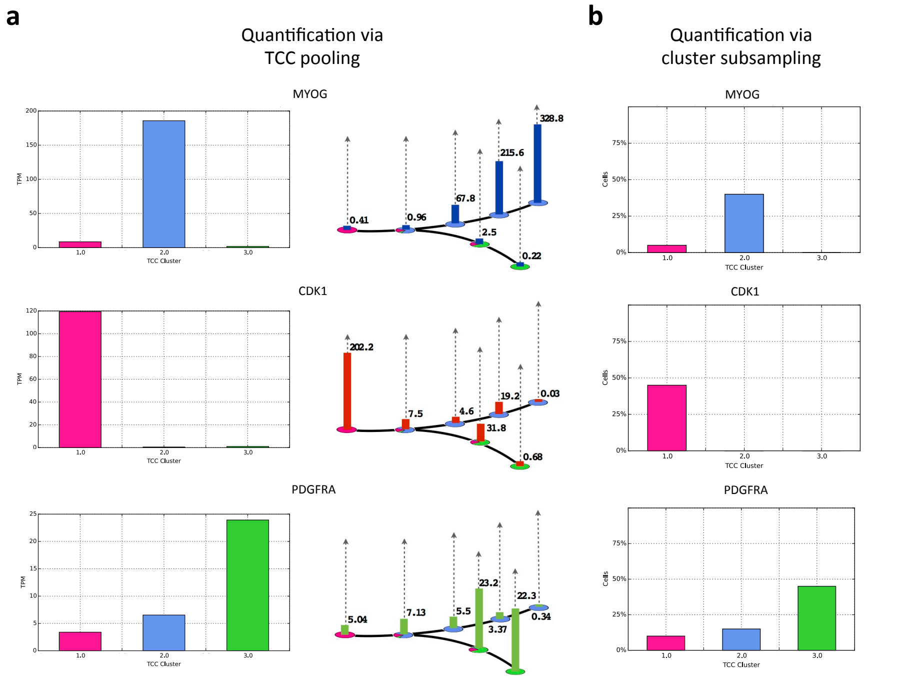

Supplementary Figure 5: Quantifying after clustering to validate clusters obtained. (a) The expression levels obtained after running kallisto's EM algorithm on the pooled TCCs of each cluster. The left pane shows the mean TPMs of the 3 clusters. The right pane shows the mean TPMs of the 7 clusters overlaid on the MST from Figure 4. We note that these were obtained by running the EM algorithm 3 times and 7 times, respectively (once for each cluster). We also note that the TPMs are similar to those of Figure $4d$. **(b)** Here we show an estimate of the number of cells expressing the each of 3 genes. The expression levels are obtained by randomly sampling 20 cells from each cluster and quantifying them. We note that the numbers obtained are similar to those of the middle pane Figure $4d$.

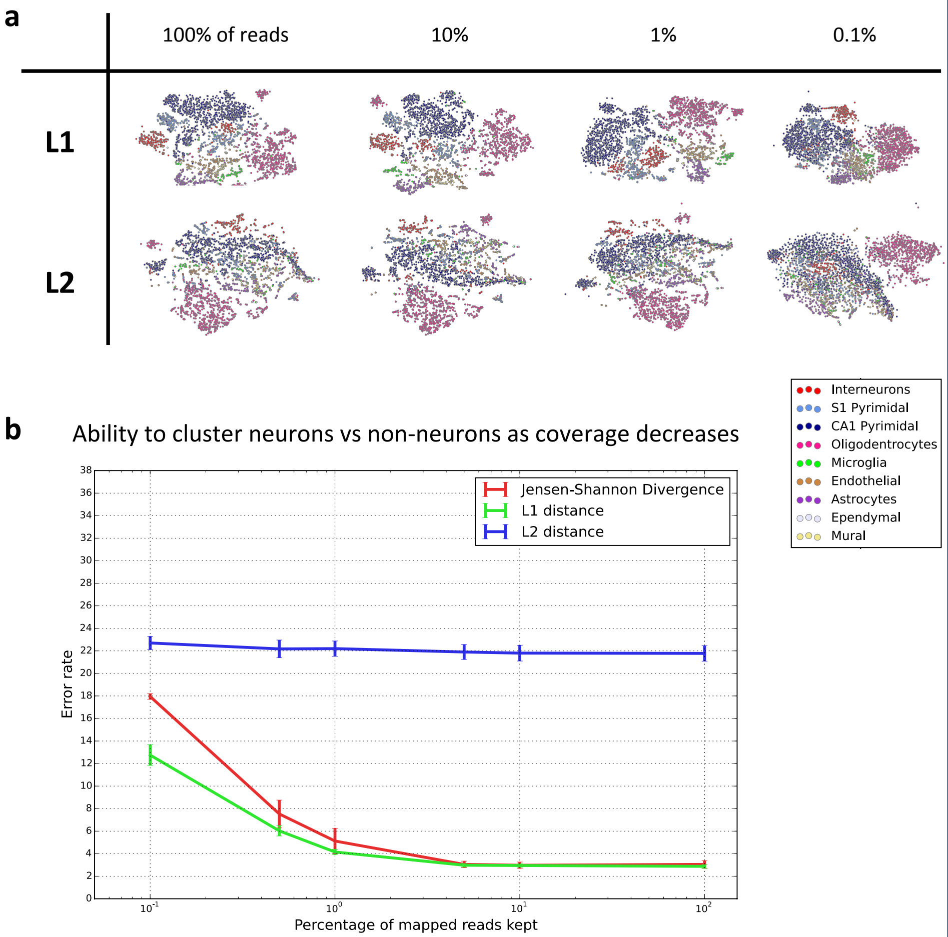

Supplementary Figure 6: Comparison of different distance metrics to use to compute pairwise distances. (a) Shows the t-SNEs obtained when using ℓ_1 (Manhattan distance or twice the total variation distance) and ℓ_2 distances (Euclidean distance) instead of Jensen-Shannon distances to compute pairwise distances between TCC histograms obtained for 3005 mouse brain cells of Zeisel *et al.* [1]. The ℓ_1 distance seems to maintain the cluster centers to a much larger extent than ℓ_2 distances. **(b)** As the average read coverage of each cell in the dataset decreases from approximately 627,000 mapped reads, spectral clusterings based on different distance metrics exhibit varying ability to distinguish neurons from non-neurons. While both ℓ_1 distance and Jensen-Shannon Divergence perform similarly well at high coverage (error rate 5%), the commonly used ℓ_2 distance resulted in significantly worse performance. We note that ℓ_2 distance is known to be a bad metric to use while comparing probability distributions. For the two classes picked, ℓ_1 distances perform better that Jensen-Shannon distance at low coverage.

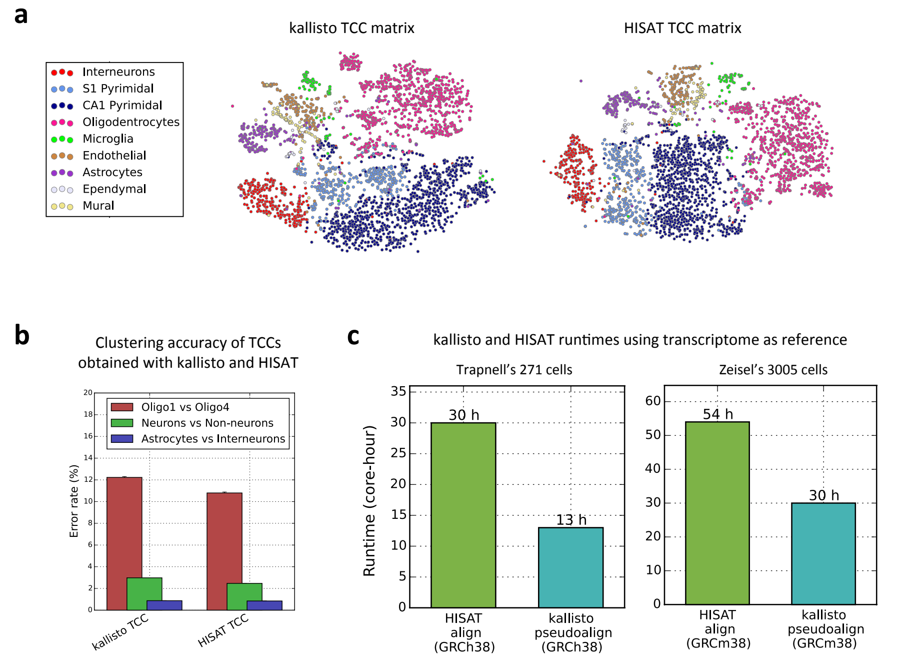

Supplementary Figure 7: Comparing alignment-based TCC with pseudoalignment-based TCC Alignment was performed using HISAT on the mouse transcriptome (GRCm38) in the case of Zeisel's dataset and the human transcriptome (GRCh38) in the case of Trapnell's dataset. HISAT's `--no-spliced-alignment` option was used. TCC vectors can be generated from aligned reads by simply counting the number of ambiguous reads aligned to each set of transcripts. For Zeisel et al.'s dataset, HISAT maps $1,843,467,887$ reads to $417,515$ equivalence classes, and kallisto maps $1,768,321,229$ reads to $246,981$ equivalence classes. We compare the **(a)** t-SNE visualizations on Zeisel et al.'s dataset, **(b)** clustering accuracies on Zeisel et al.'s dataset, and **(c)** runtimes of the two approaches on both Zeisel and Trapnell et al.'s datasets. Overall, alignment-based TCCs yield slightly better cell-type classification error rates on the Zeisel et al.'s dataset – at the cost however of a higher computation time.

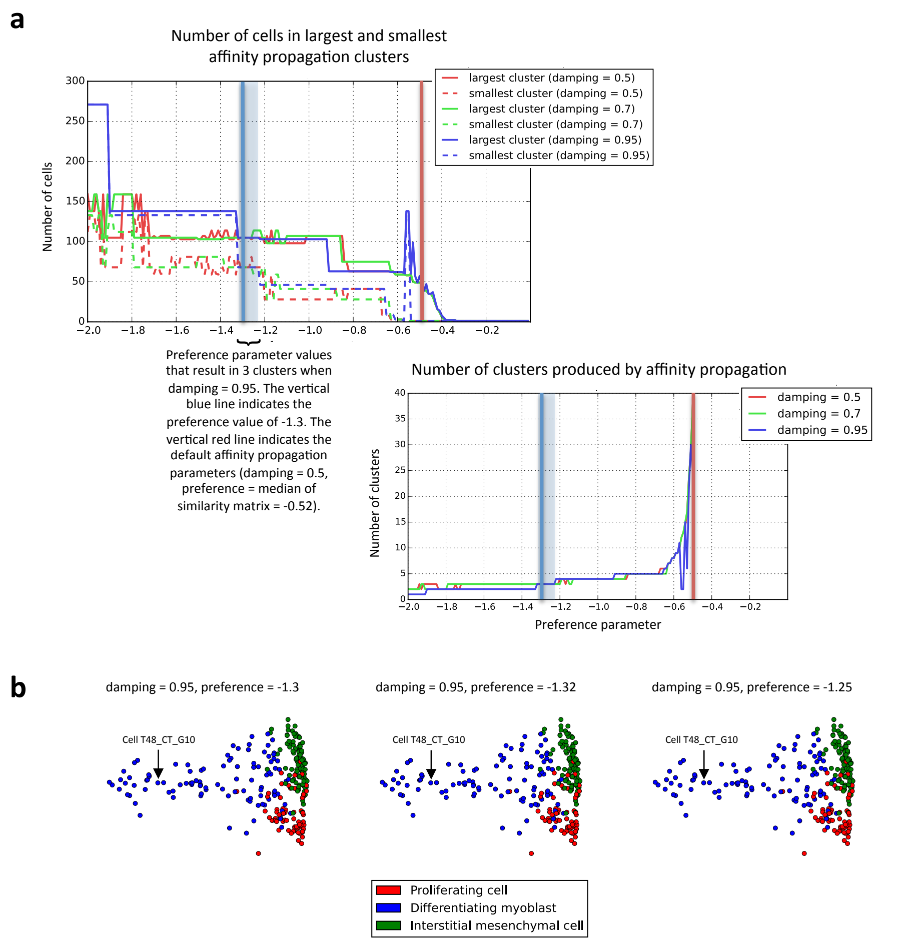

Supplementary Figure 8: Selecting parameters for affinity propagation on TCC vectors for Trapnell et al.'s data-set. (a) For the TCC approach, we performed the same parameter sweep presented in Supplementary Figure 6, resulting in the plots shown here. Additionally, we highlight the area of the curves where affinity propagation with a damping value of 0.95 results in 3 clusters. The default affinity propagation parameters of 0.5 for damping and -0.52, the median of the similarity matrix, for preference results in 24 clusters, 12 of which have only 1 member. **(b)** To test the stability of the clusters across a flat region of the curves, we looked at 3 combinations of parameters that resulted in 3 clusters when the damping value equals 0.95. The clusterings are identical, and we see that Cell T48_CT_G10 is consistently classified as a differentiating myoblast.

References

1. Zeisel, A., Muñoz-Manchado, A.B., Codeluppi, S., Lönnerberg, P., La Manno, G., Jurèus, A., Marques, S., Munguba, H., He, L., Betsholtz, C., Rolny, C., Castelo-Branco, G., Hjerling-Leffler, J., Linnarsson, S.: Cell types in the mouse cortex and hippocampus revealed by single-cell RNA-seq. Science **347**(6226), 1138–1142 (2015). doi:10.1126/science.aaa1934. http://www.sciencemag.org/content/347/6226/1138.full.pdf
2. Trapnell, C., Cacchiarelli, D., Grimsby, J., Pokharel, P., Li, S., Morse, M., Lennon, N.J., Livak, K.J., Mikkelsen, T.S., Rinn, J.L.: The dynamics and regulators of cell fate decisions are revealed by pseudotemporal ordering of single cells. Nat Biotech **32**(4), 381–386 (2014). doi:10.1038/nbt.2859
